# Quantitative analysis of fertilizer using laser-induced breakdown spectroscopy combined with random forest algorithm

**DOI:** 10.3389/fchem.2023.1123003

**Published:** 2023-01-13

**Authors:** Lai Wei, Yu Ding, Jing Chen, Linyu Yang, Jinyu Wei, Yinan Shi, Zigao Ma, Zhiying Wang, Wenjie Chen, Xingqiang Zhao

**Affiliations:** ^1^ Jiangsu Key Laboratory of Big Data Analysis Technology, Nanjing University of Information Science and Technology, Nanjing, China; ^2^ Jiangsu Collaborative Innovation Center on Atmospheric Environment and Equipment Technology, Nanjing University of Information Science and Technology, Nanjing, China; ^3^ School of Automation, Nanjing University of Information Science and Technology, Nanjing, China

**Keywords:** LIBS, fertilizer, RF, PLS, pH

## Abstract

Chemical fertilizers are important for effectively improving soil fertility, promoting crop growth, and increasing grain yield. Therefore, methods that can quickly and accurately measure the amount of fertilizer in the soil should be developed. In this study, 20 groups of soil samples were analyzed using laser-induced breakdown spectroscopy, and partial least squares (PLS) and random forest (RF) models were established. The prediction performances of the models for the chemical fertilizer content and pH were analyzed as well. The experimental results showed that the *R* ^2^ and root mean square error (RMSE) of the chemical fertilizer content in the soil obtained using the full-spectrum PLS model were .7852 and 2.2700 respectively. The predicted *R* ^2^ for soil pH was .7290, and RMSE was .2364. At the same time, the full-spectrum RF model showed *R* ^2^ of .9471 (an increase of 21%) and RMSE of .3021 (a decrease of 87%) for fertilizer content. *R* ^2^ for the soil pH under the RF model was .9517 (an increase of 31%), whereas RMSE was .0298 (a decrease of 87%). Therefore, the RF model showed better prediction performance than the PLS model. The results of this study show that the combination of laser-induced breakdown spectroscopy with RF algorithm is a feasible method for rapid determination of soil fertilizer content.

## 1 Introduction

Soil is an indispensable part of the living environment of plants (Palansooriya et al., 2020) and a natural resource necessary for human survival (Zhang et al., 2018). Soil environment is directly related to the survival of plants and human beings (Gondek et al., 2018) and affects agricultural production (Hou et al., 2019). Crops cannot be planted without soil, and the fertility of the soil determines the growth and development of crops, which directly affects the yield and quality of the crops. Therefore, effective soil fertilization can promote sustainable development of agriculture and improve planting efficiency.

In modern agriculture, the use of chemical fertilizers is very prevalent. For example, ferrous sulfate fertilizer can not only supplement iron in plants, but also promote the absorption of nitrogen and phosphorus. Because ferrous sulfate has strong reducibility, it can also greatly regulate the oxidation-reduction process in plants. However, during fertilization, some areas receive excessive fertilizer, resulting in imbalances in the soil nutrient structure, worsening of the physical properties of soil, and concentrations of harmful metals and bacteria exceeding the standard values, which affects crop production (Guo et al., 2022). For example, long-term excessive use of nitrogen fertilizers (Zhao et al., 2022) leads to the loss of calcium, magnesium, and other elements, resulting in a continuous soil acidification and eventual loss of productivity. Excessive use of potassium fertilizers hinders the growth of crops, leading to lodging and other crop symptoms, reduction in crop production, and weakening of the production capacity of crops. At the same time, insufficient application of chemical fertilizers leads to insufficient soil fertility and reduced crop yield. Therefore, it is necessary to determine the contents of chemical fertilizers in soil.

At present, the contents of chemical fertilizers in soil are determined mainly through the potential method. Although this method has the advantages of accuracy and high precision (Topcu and Isildak, 2021), large-scale soil analysis is difficult because of the complicated pretreatment process and long analysis time. Therefore, it is necessary to develop a rapid and effective method for the determination of fertilizer content in soil. Laser-induced breakdown spectroscopy (LIBS) (Ding et al., 2021) is a new atomic emission spectroscopy technology with laser as the excitation source. Compared with the traditional analysis method, LIBS has the advantages of rapidness, real-time assessment (Ding et al., 2020), on-site micro-loss analysis (Qi et al., 2018), remote detection (Agresti et al., 2022), no need for complex sample preparation (Gupta et al., 2020), and simultaneous analysis of multiple elements (Shukla et al., 2022). In recent years, LIBS has been successfully used in geological exploration (Rethfeldt et al., 2021), metallurgical analysis (Myakalwar et al., 2021), medical diagnosis (Alsharnoubi et al., 2020), archaeology (Wallace et al., 2020), environmental monitoring (Ding et al., 2019), and other fields, including fertilizer detection. Danie et al. converted liquid fertilizer into a solid and used LIBS to analyze Cu, K, Mg, Mn, Zn, As, Cd, Cr, and Pb contents in the fertilizer, with a detection error of .02%–.06% (Andrade et al., 2017); Sha. et al. combined LIBS with multiple linear regression to determine the concentration of phosphorus in compound fertilizer (SHA et al., 2018); Senesi et al. analyzed phosphate rock and organic mineral phosphate fertilizer by single-pulse and double-pulse LIBS with principal content analysis and partial least squares (PLS) algorithm, and the identification results reached a confidence level of 95% (Senesi et al., 2017). At present, LIBS primarily focuses on the detection of elements in the fertilizer and is rarely applied to measuring the content of fertilizer in soil.

The soil matrix is complex; thus, its LIBS spectral information is also complicated (Chatterjee et al., 2019), which limits the application of LIBS technology to the accurate analysis of chemical fertilizer content in soil. Chemometric methods, such as PLS (Ewusi-Annan et al., 2020), support vector machine (Yuan et al., 2020), artificial neural networks (Babu et al., 2021), and random forest algorithm (Ding et al., 2020; Ding et al., 2019), are effective tools (Musyoka et al., 2022) for accurate qualitative and quantitative analysis using LIBS (Feng et al., 2022).

In this study, LIBS combined with PLS and RF algorithms was used to analyze soil samples with added ferrous sulfate fertilizer. Particularly, LIBS was used to readily and accurately determine the amount of fertilizer, thus providing effective reference for the follow-up management of fertilized soil.

## 2 Materials and methods

### 2.1 Soil sample preparation

The soil samples were obtained from a farmland in Yangzhong, Jiangsu Province. Twenty samples with different concentration gradients were prepared by adding different amounts of fertilizer to the soil. The concentration gradient and pH of the samples are listed in Table 1. In this experiment, ferrous sulfate fertilizer was used, and according to the amount of fertilizer, different quantities of the ferrous sulfate fertilizer were weighed, then added to 30 ml of distilled water, and stirred evenly. The prepared solutions were uniformly mixed with 100 g of the soil, put in an oven, and baked at 150°C for 10 h. The baked samples were then ground into powder; 2 g samples of soil powder were weighed, put into a tablet press, and pressed for 3 min at 20 MPa, yielding samples with a final diameter of 13 mm and a thickness of 5 mm.

**TABLE 1:** Concentration of ${FeSO}_{4}$ (Wt%).

Sample number	Concentration (mg/g)	pH
1	0	6.2
2	6	6.15
3^a^	9	6.12
4	12	6.09
5	15	6.07
6	18	6.04
7	21	6.02
8^a^	24	6.00
9	27	5.98
10	30	5.94
11^a^	33	5.90
12	36	5.86
13	39	5.82
14	42	5.79
15^a^	45	5.75
16	48	5.72
17	51	5.69
18	54	5.65
19	57	5.61
20	60	5.58

^a^ represents the test set.

### 2.2 LIBS setup

**FIGURE 1:**
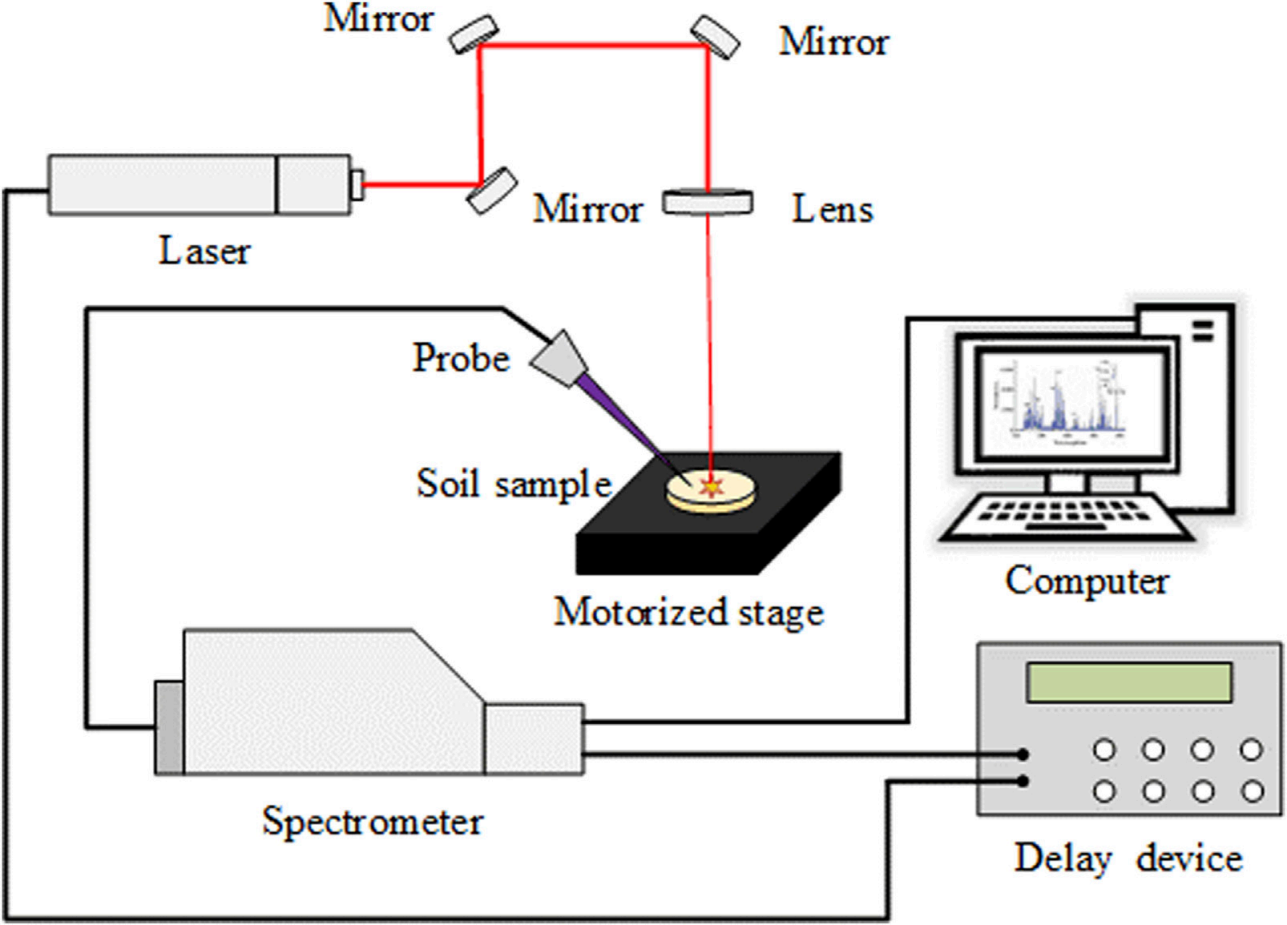
Experimental setup.

A lamp-pumped electro-optic Q-switched compact nanosecond laser (Beamtech China, Dawa-200) was used as the excitation source (Figure 1). A wavelength of 1,064 nm, working frequency of the laser of 1 Hz, pulse energy of 55 mJ, spectral integration time of 1.05 m, and delay time of 3 μs were used in this study. During the experiment, the soil sample was placed directly on the moving stage, and the high-energy laser pulse was focused on the surface of the sample through a focusing mirror (with a focal length of 100 mm), which ablated the sample and generated plasma. Subsequently, the optical fiber probe collected the radiated spectral signal and coupled it to the spectrometer (Avantes AvaSpec-ULS2048-2-USB2, .07 nm). The wavelength range was 198–425 nm, with a total of 4,096 wavelength points.

To reduce the fluctuation of the LIBS spectrum caused by the non-uniformity of the samples, 20 measurement points were randomly selected for each sample. Each measurement point was analyzed 3 times, and an average spectrum was recorded. Finally, 20 analysis spectra were obtained and averaged into one spectrum for each sample.

### 2.3 Methods

#### 2.3.1 PLS

PLS is a regression-based mathematical optimization algorithm for multiple dependent and independent variables. In this method, the linear regression equation between independent and dependent variables is established, principal components are extracted step by step from the two matrices, variance and covariance are calculated, and iteration is performed one step at a time. Finally, according to the cross-validation results, the quantitative regression prediction and PLS analysis model are established (Alvarez et al., 2022). Conceptually, PLS primarily consists of extracting the first principal component from the independent variable and dependent variable matrix, and obtaining the covariance; then the second principal component is extracted and covariance is obtained, and this process is iterated. Finally, according to the cross-validation results, the final PLS quantitative regression prediction analysis model is established (Garcia et al., 2021). This algorithm can overcome the problem of collinearity between multiple independent variables.

#### 2.3.2 RF

RF is a classifier that contains multiple decision trees, and the output is determined by the mode of each tree’s output category. RF is a machine learning method (AGMD et al., 2021) which generates a large number of decision trees through randomly selected training samples and variable subsets, and uses these decision trees to predict the results to avoid over-fitting (Zagajewski et al., 2021). Compared with other traditional algorithms, RF has a higher calculation speed and stronger generalization ability and thus a lower risk of over-fitting (Gk et al., 2021).

## 3 Results and discussion

### 3.1 Analysis of LIBS spectra

**FIGURE 2:**
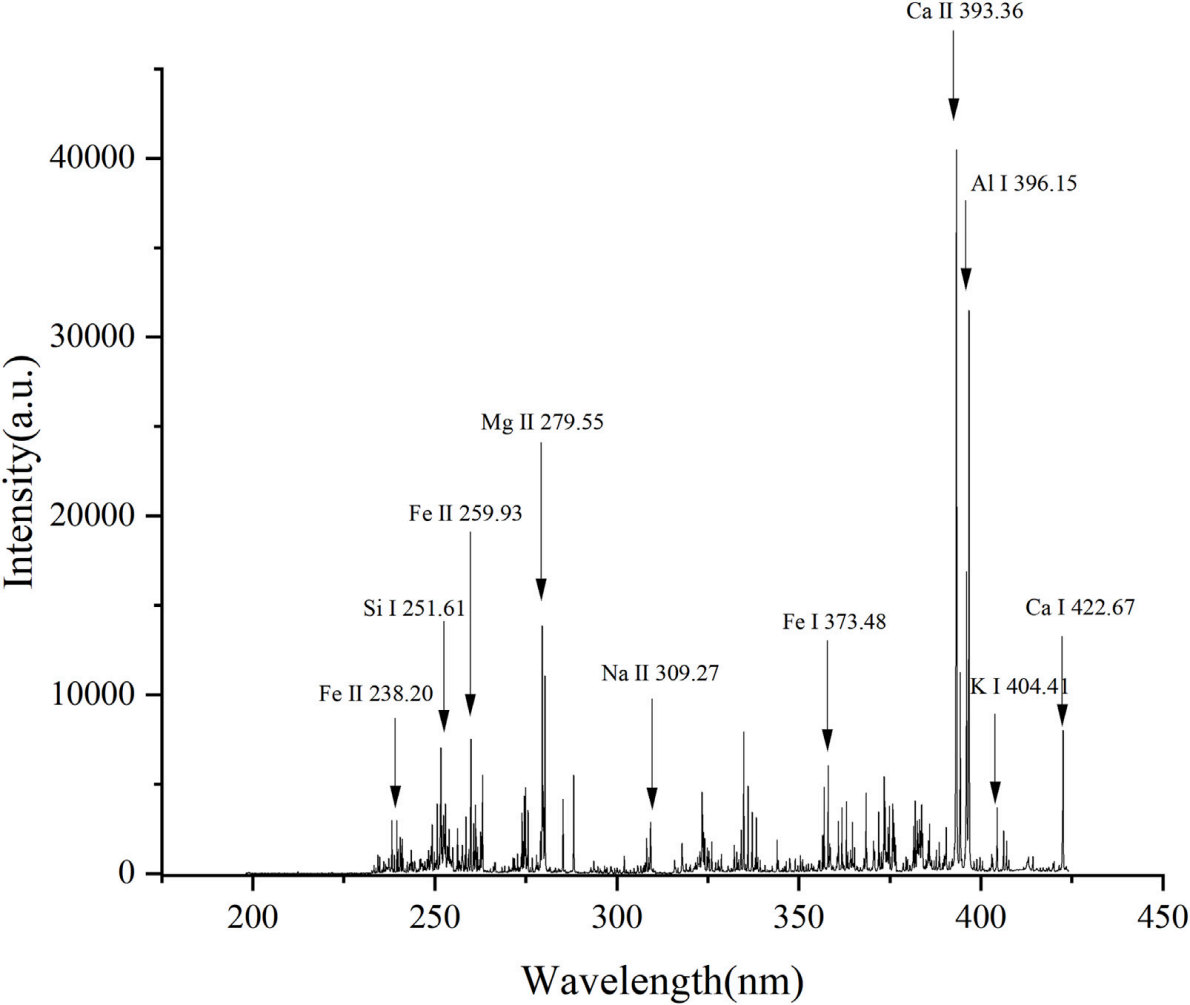
LIBS spectrum of soil samples.

Wavelength and intensity are important factors in the qualitative analysis of soil elemental content. Figure 2 shows the average LIBS spectra of all soil samples, and the elements with strong characteristic lines are marked according to the NIST database. As shown in Figure 2, Fe, Al, Ca, Mg, and Si are the main elements in soil, with the characteristic spectral lines of Fe Ⅱ (238.20 nm), Al Ⅰ (396.15 nm), Ca Ⅱ (393.36 nm), Mg Ⅱ (279.55 nm), and Si Ⅰ (251.61) nm.

### 3.2 PLS model prediction performance

**FIGURE 3:**
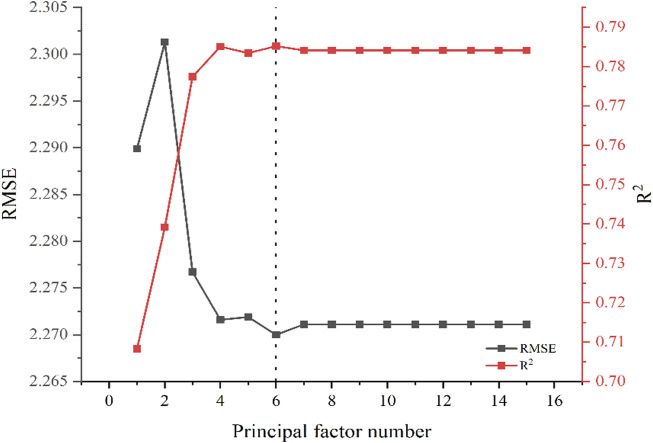
The relationship between principal factor number and R^2^/RMSE.

Before the prediction, the PLS method was optimized. The principal factor number is an important parameter of the PLS model. To avoid insufficient fitting or over-fitting of the model, the principal factor number must be optimized. Thus, based on spectral data, PLS quantitative analysis models for soil fertilization content with 1–15 principal factors were established, and the curve fitting coefficient *R* ^2^ and root mean square error (RMSE) predicted by the models were analyzed. The results are shown in Figure 3. *R* ^2^ gradually increases with the increase in the principal factor number from one to 4. With a further increase in the principal factor number, *R* ^2^ fluctuates and reaches the maximum value of .7852 when the principal factor number is 6. RMSE is at its maximum when the principal factor number is 2. At 6 principal factors, RMSE shows the minimum value of 2.2700. *R* ^2^ and RMSE exhibit best values at six principal factors; thus, six was selected as the optimum principal factor number in this study. Therefore, the full-spectrum data for six factors was used to predict and analyze the content of fertilizer in soil. With the increase in soil fertilization, the pH of the soil changes to a certain extent; as this change impacts crop growth, LIBS was also used to explore soil pH in this study.

**FIGURE 4:**
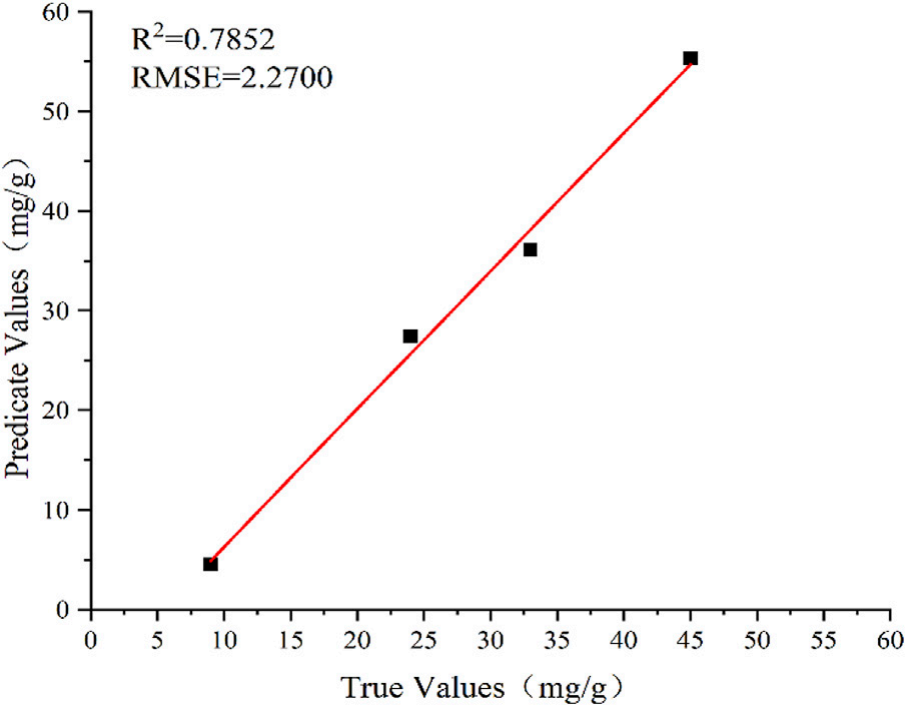
Prediction results using PLS model for fertilization.

**FIGURE 5:**
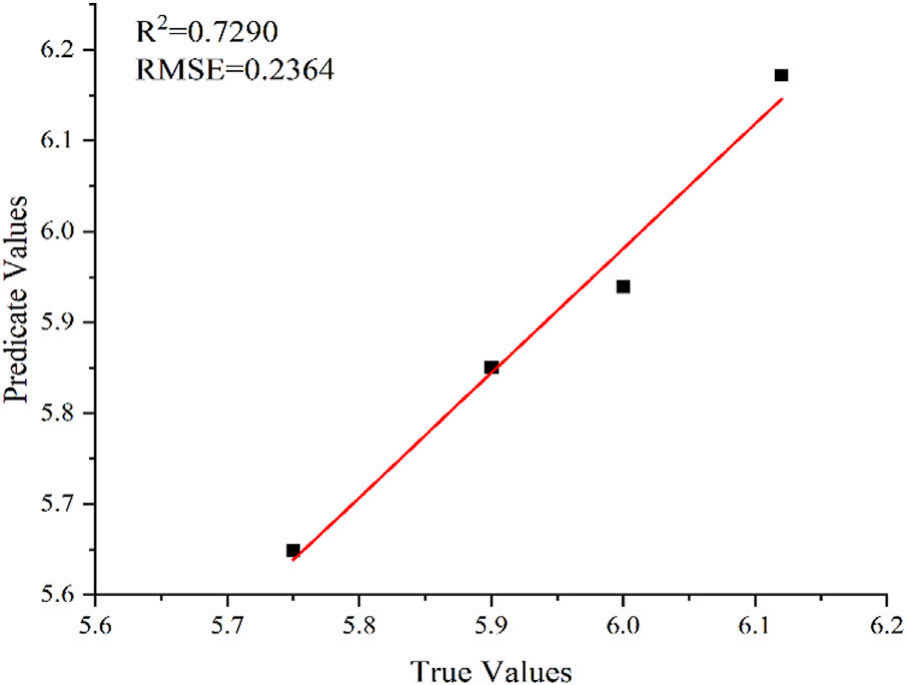
Prediction results using PLS model for/pH.

In this work, the full spectrum was used to predict the amount of fertilizer. Using the full-spectrum information with 4,096 spectral points as input variables, a PLS model was established to predict the amount of fertilizer. The prediction results are shown in Figure 4. Under the PLS model, the *R* ^2^ and RMSE of the fertilizer content predicted using the full spectrum of soil are .7852 and 2.2700, respectively. The PLS model based on full-spectrum data was then used to predict the soil pH (Figure 5). The *R* ^2^ and RMSE of pH in the test set are .7290 and .2364, respectively.

The results show that the accuracy of the PLS model in predicting the fertilizer content and soil pH are not satisfactory. The correlations between the actual and predicted values of the fertilizer content and pH are poor. The analysis error may be caused by excessive interference information in the soil spectrum, which had a negative impact on the prediction results of the model, resulting in the poor fitting of the PLS model, limited prediction performance, and failure to obtain good prediction results.

### 3.3 RF model prediction performance

**FIGURE 6:**
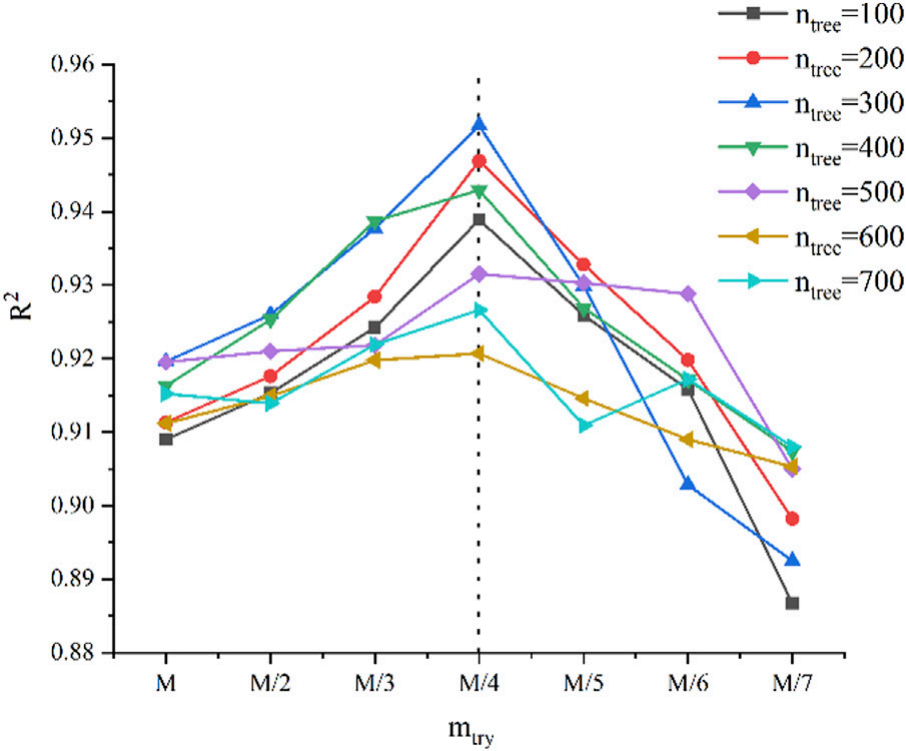
Prediction of R^2^ with different values of *n*_tree_ and *m*_try_.

In the RF model, the main parameters affecting the model are *n*_tree_ and *m*_try_; *n*_tree_ is the number of decision trees, and *m*_try_ specifies the number of variables used in binary trees in nodes and the number of variables affecting the data sets. In this experiment, we optimized the model by comparing the changes in *R* ^2^ and RMSE predicted by the models with different *n*_tree_ and *m*_try_. Algorithm optimization was performed for *n*_tree_ values in the range of 100–700, with the increments of 100. The *m*_try_ values were *M*, *M*/2, *M*/3, *M*/4, *M*/5, *M*/6, and *M*/7, where *M* is the total number of variables. The optimization process is shown in Figure 6. *R* ^2^ gradually increases with *n*_tree_ until it reaches the maxima at *n*_tree_ = 300, then gradually decreases, and stabilizes, whereas *m*_try_ gradually increases in the change from *M* to *M*/4 and then gradually decreases in the variation from *M*/4 to *M*/7. Therefore, at the *n*_tree_ and *m*_try_ of 300 and *M*/4, respectively, the model results are the best. The RF model was built based on the input variables and optimized model parameters, and the full spectrum was used to predict the fertilizer content and soil pH.

**FIGURE 7:**
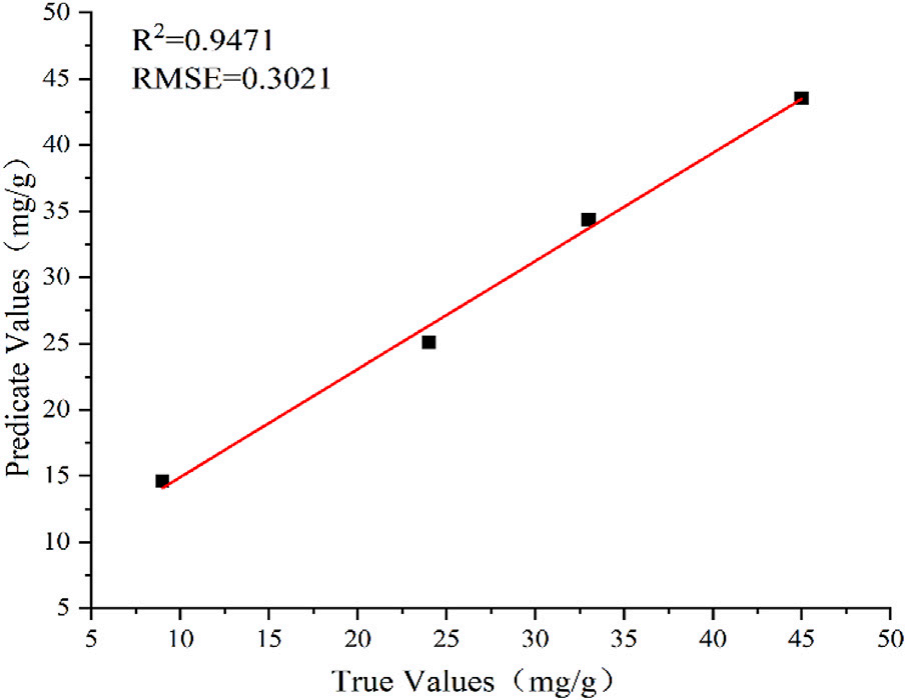
Prediction results using RF model for Fertilization.

**FIGURE 8:**
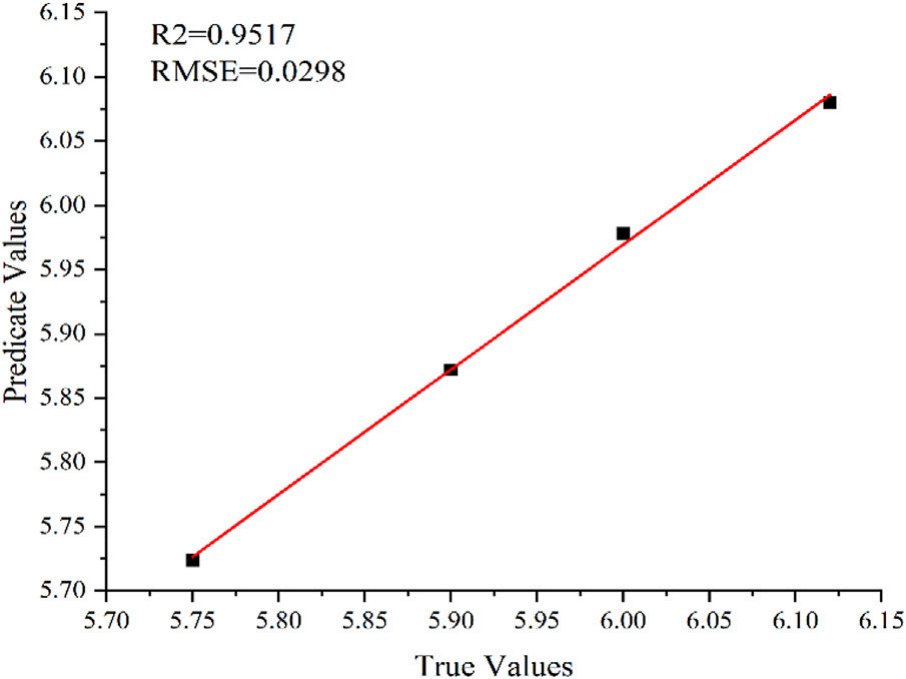
Prediction results using RF model for pH.

All spectral points measured by LIBS were imported into the RF data model, and the prediction results are shown in Figure 7 (*R* ^2^ = .9471 and RMSE = .3021). The RF model was also used to predict the soil pH based on full spectrum. The prediction results are shown in Figure 8 (*R* ^2^ = .9517 and RMSE = .0298). Therefore, the optimized RF model shows good prediction results for the fertilizer content and soil pH.

### 3.4 Comparison of prediction performance of the PLS an RF models

The comparison results of prediction performance is listed in Table 2. The results of the optimized PLS model (6 principal factors) for the full-spectrum prediction of fertilizer content are unsatisfactory. At the same time, the prediction accuracy of the RF model is considerably better: *R* ^2^ is higher by 21% than that obtained using PLS (.9471 and .7853, respectively), and RMSE is lower by 87% (2.2700 and .3021, respectively). Thus, the RF model demonstrates better accuracy and stability of the prediction results. For the full-spectrum-based prediction of soil pH, *R* ^2^ obtained by the RF model is higher by 31% than that in the PLS model (.9517 and .7290, respectively), whereas RMSE is lower by 87% (.0298 and .2364, respectively). Therefore, the RF prediction model also showed higher accuracy than the PLS model in predicting soil pH.

**TABLE 2:** Prediction results of two algorithm models

Algorithm	Model established	R^2^	RMSE
PLS	Fertilizer	0.7852	2.2700
	pH	0.7290	0.2364
RF	Fertilizer	0.9471	0.3021
	pH	0.9517	0.0298

Thus, the optimized RF model is significantly better than the optimized PLS model in predicting fertilizer content and soil pH based on the full-spectrum data. The combination of LIBS with the RF method is effective and feasible for analyzing the amount of fertilizer in the soil and soil pH.

## 4 Conclusion

The main purpose of this study was to use LIBS in combination with PLS and RF methods to analyze soil samples with added ferrous sulfate fertilizer. Herein, the RF and PLS methods were used to predict the amount of fertilizer and pH of soil samples. The experimental results demonstrated that the *R* ^2^ and RMSE for the prediction of fertilizer content in soil using the PLS model based on full spectrum were .7852 and 2.2700, respectively. The predicted *R* ^2^ of soil pH was .7290 and RMSE was .2364. At the same time, the full-spectrum RF model showed the predicted *R* ^2^ of .9471 (higher by 21%) and RMSE of .3021 (lower by 87%) for the fertilizer content; furthermore, for the soil pH, the predicted *R* ^2^ was .9517 (higher by 31%), and RMSE was .0298 (lower by 87%). Thus, the RF model showed better prediction performance than the PLS model. Therefore, the combination of LIBS with the RF algorithm is a feasible method for determining the amount of fertilizer in the soil and the soil pH. The methods studied herein provide effective reference for the follow-up management of fertilized soil.

## Data Availability

The raw data supporting the conclusion of this article will be made available by the authors, without undue reservation.
